# Supplementary material for: Gingival Necrosis Related to Sepsis-Induced Agranulocytosis Due to Pseudomonas aeruginosa Bacteraemia: A Case Report
Source: J Clin Med. 2024 Feb 24;13(5):1285. doi: 10.3390/jcm13051285 (PMC10931707; doi:10.3390/jcm13051285)
Supplement: Supplementary file 1 [file jcm-13-01285-s001.zip › jcm-2850117-supplementary.pdf]

# Gingival necrosis related to sepsis-induced agranulocytosis due to Pseudomonas aeruginosa bacteraemia: A case report

Jia Ying Tan, Guo Nian Teo, Ethan Ng, Andrew Ban Guan Tay, John Rong Hao Tay

## Content of the Supplementary

Figure S1. Initial clinical presentation of the patient’s gingiva undergoing necrosis.

Figure S2. Erect anteroposterior chest radiograph.

Figure S3. Contrast-enhanced computed tomography scan of the abdomen and pelvis.

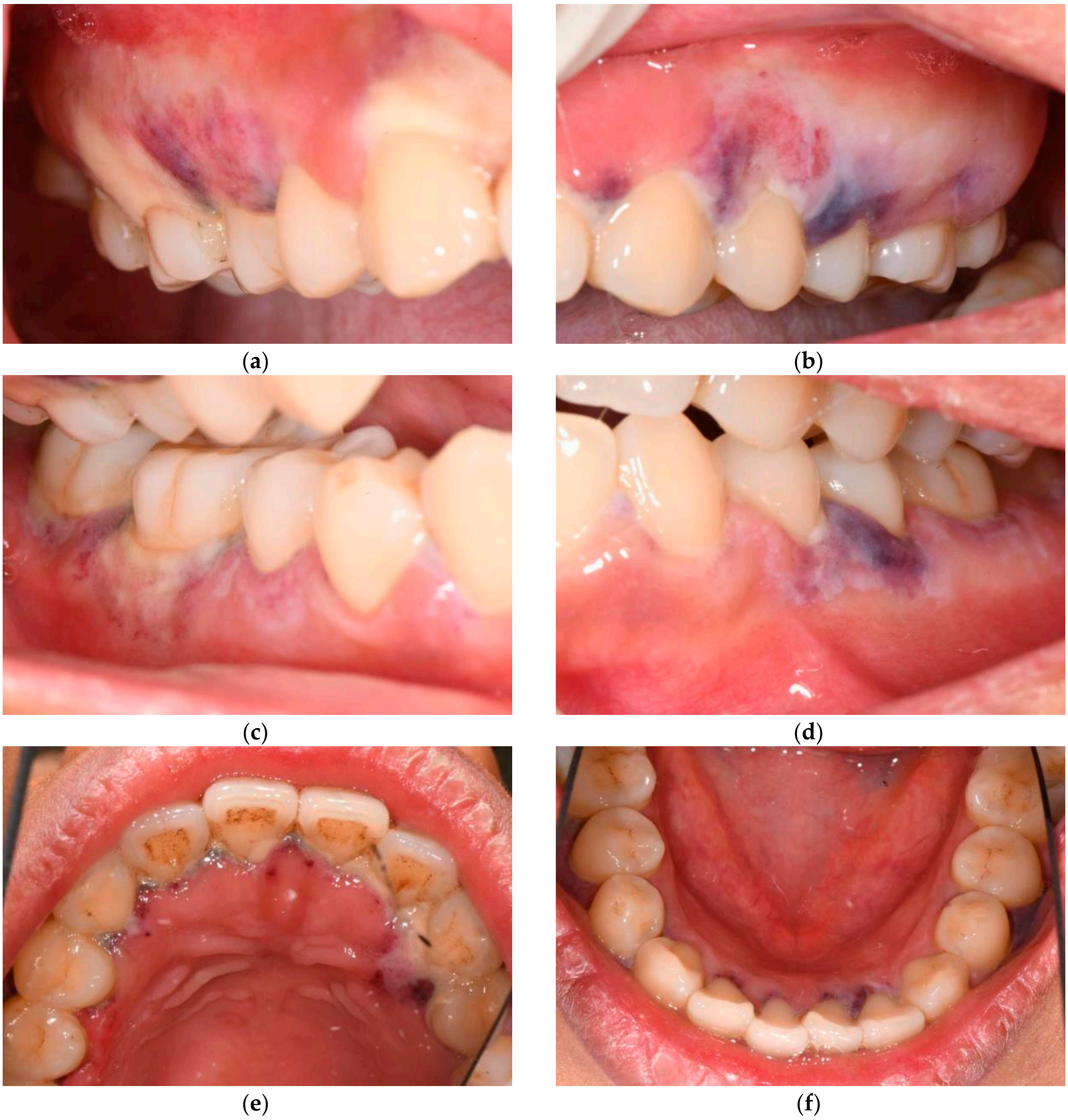

Figure S1**.** Initial clinical presentation of the patient's gingiva undergoing necrosis. Varying degrees of purplish-pink patches and white necrosis noted. Purple purpura-like appearance seems to be a precursor to the white necrotic appearance. **(a)** Buccal of upper right quadrant. **(b)** Buccal of upper left quadrant. **(c)** Buccal of lower right quadrant. **(d)** Buccal of lower left quadrant. **(e)** Palatal of upper anteriors. **(f)** Lingual of lower anteriors.

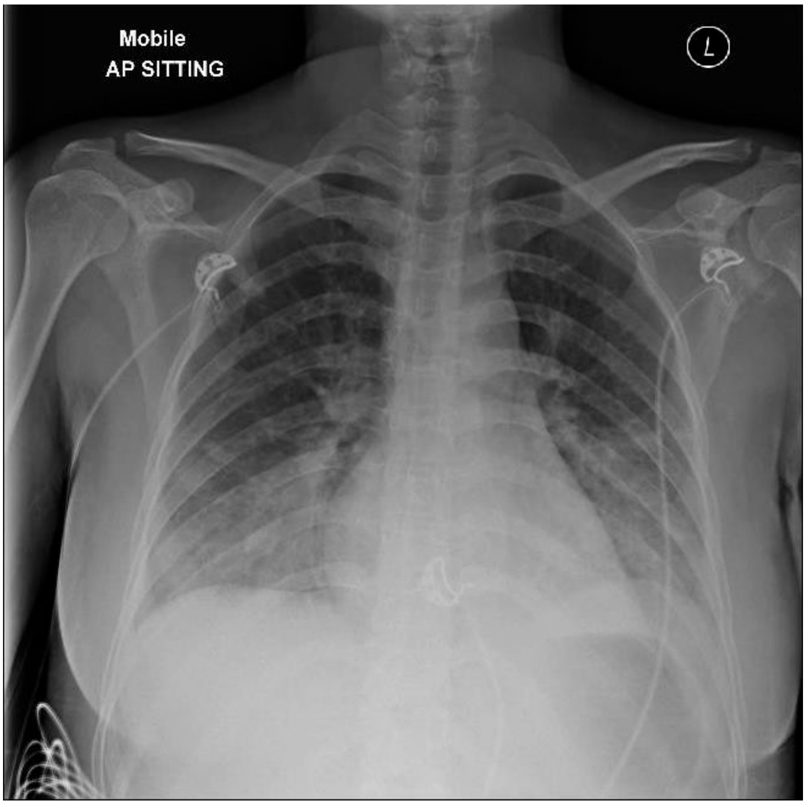

Figure S2. Erect anteroposterior chest radiograph showing consolidation in the lower lobes of the lungs.

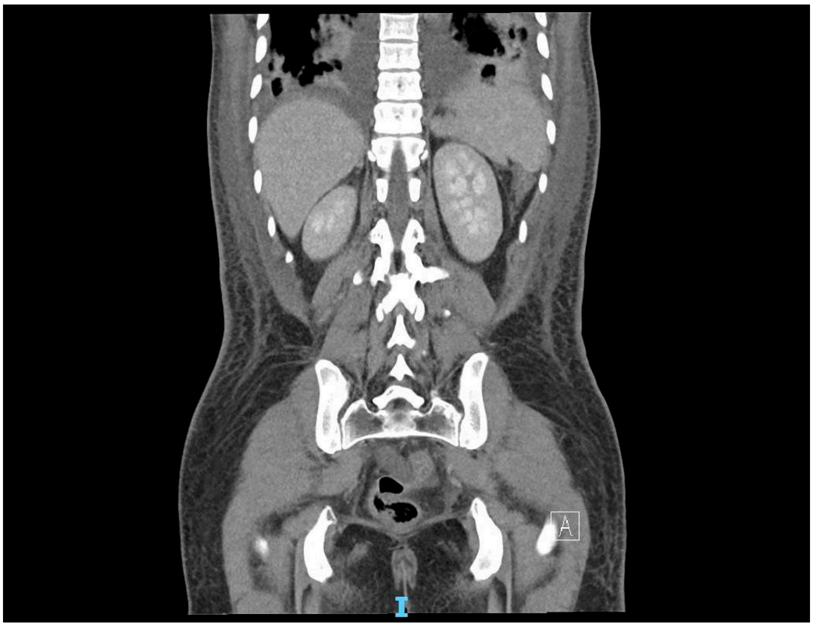


(**a**)

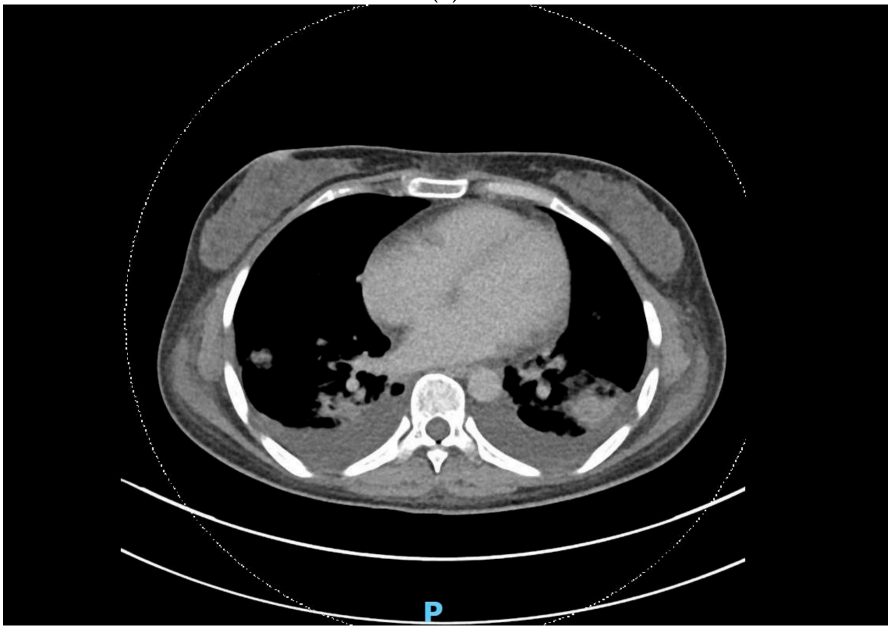


(**b**)

**Figure S3.** Contrast-enhanced computed tomography scan of the abdomen and pelvis. No source of sepsis is noted in the abdomen and pelvis. Lung bases show small bilateral pleural effusions. Air-space consolidation is seen in the lower lobes of the lungs, and in the left lingula, representing infection (pneumonia). **(a)** Coronal view. **(b)** Axial view.
